# MetaOntology: Toward developing an ontology for the metaverse

**DOI:** 10.3389/fdata.2022.998648

**Published:** 2022-09-08

**Authors:** Bilal Abu-Salih

**Affiliations:** ^1^King Abdullah II School of Information Technology, The University of Jordan, Al Jubeiha, Jordan; ^2^School of Management and Marketing, Curtin University, Perth, WA, Australia

**Keywords:** metaverse, metaverse ontology, virtual reality, augmented reality, extended reality

## Abstract

Metaverse is now perceived as a celebrated future version of the internet. In this new anticipated virtual universe, interconnected digital platforms leveraged by augmented, extended, and virtual realities will elevate users' immersive experiences through multidimensional interactions. In particular, users will be offered a broad spectrum of digital activities within a newly immersive setting mediated by technology. This study aims to design a domain ontology (MetaOntology) for the metaverse to provide an explicit specification of relevant state-of-the-art technologies and infrastructure. A four-step methodological approach is followed to construct the designated ontology. Due to the immaturity of the metaverse, MetaOntology is not intended to furnish a complete outlook on the domain, rather it aims to establish a cornerstone so as to facilitate future efforts in building extant versions of this ontology considering the evolvement of relevant technologies.

## Introduction

The Metaverse has recently witnessed significant momentum, notably after Facebook rebranded to *Meta*. Although this portmanteau term was coined decades ago when indicated in the hit novel “Snow Crash” by Neal Stephenson, there is no consensus on its definition. This is caused by the metaverse currently being developed and is relatively new to define what it means. For instance, the internet existed in the 1970s, but not all preconceived notions about what it would eventually look like were accurate. Yet the current discussions about the metaverse perceive it as a single physical and digital space connected through representative virtual reality and augmented reality technologies (Kim W. -S, 2021; Suh and Ahn, 2022; Yang et al., 2022).

Users in this new ecosystem will be able to access 3D virtual or augmented reality environments using gadgets like virtual reality headsets, digital glasses, smartphones, and other devices. In these environments, they will be able to work, communicate with friends, conduct commerce activities, travel to distant locations, and take advantage of educational opportunities—all within a newly immersive setting and mediated by technology. More mergers and synergies might occur as technology converges. It is conceivable that the metaverse definition will encompass new fields and prospective development areas. Therefore, there is a need to define a common vocabulary, terminology, and modeling representations of this new domain, including dimensions of state-of-the-art technologies, thereby obtaining a better understanding of the structure of information currently propagated in this innovative ecosystem. To capture the current domain representation of the metaverse, *ontology engineering* must be incorporated.

Ontology engineering entails developing a thorough and exact conceptual framework for a particular domain. This is leveraged by means of Ontology and Semantic Web technology. Gruber (1993) defined ontology as an explicit formal specification of a conceptualization. In order to offer a formal representation in machine-understandable semantics, ontology captures the domain knowledge through the defined concrete concepts (representing a collection of entities), constraints (rules), and the relation between concepts. Building ontologies enables automated machine validation and verification of applications and services and facilitates information sharing, reusing, and reasoning (Miksa and Rauber, 2017). Ontology also integrates heterogeneous data sources and facilitates interoperability between different data models (Wongthontham and Abu-Salih, 2018; Abu-Salih et al., 2021). Similar to how it occurs in the real world, interoperability is essential to link various metaverse projects so that users can enjoy a consistent experience while participating in various socio-cultural activities. Interoperability and decentralized technologies will strengthen the metaverse (Ning et al., 2021). Without interoperability, the metaverse will continue to be overloaded, preventing widespread adoption, especially among the commerce and banking sectors, which depend on an interoperable infrastructure to run their daily operations (Kim J, 2021). Thus, developing an ontology can offer a seamless extensibility and support interoperability (Bassiliades et al., 2018) which is deemed necessary for the metaverse (Valaskova et al., 2022; Xu et al., 2022). Further, through the facilitation of knowledge capture, storage, integration, and query, ontology technology may enhance coordination and interaction across various metaverses and metaverse applications. Despite limited efforts to offer taxonomies for the metaverse in terms of its infrastructure (Lee et al., 2021; Park and Kim, 2022; Seidel et al., 2022), to the best of our knowledge, there has not been any attempt to create an ontology for the metaverse domain devoted to standardizing and formalizing the state-of-the-art technologies for the designated domain knowledge.

This study aims to design a domain ontology (MetaOntology) for the metaverse to provide an explicit specification and a better understanding of relevant state-of-the-art technologies and infrastructure. This study follows Design Science Research Methodology (DSRM) to construct the appropriate artifacts. Further, we follow a methodological approach to construct MetaOntology. This methodology integrates two ontology development techniques, namely METHONTOLOGY (Fernández-López et al., 1997) and Cyc 101 (Lenat and Guha, 1993). In particular, a four-step process is followed to design, implement, and evaluate MetaOntology: (i) determine the domain and scope of the ontology; (ii) ontology reuse; (iii) conceptual model development; and (iv) ontology evaluation. The metaverse domain is still immature. Hence, our constructed ontology is not intended to furnish a complete outlook on the domain, rather it aims to build a cornerstone so as to facilitate future efforts in building extant versions of this ontology considering the evolvement of new sophisticated advancements.

Section Related works discusses recent works in ontology design to benefit VR applications. Section Research methodology presents the research methodology followed in this study. Section MetaOntology: Ontology design for the metaverse details the design, implementation and evaluation of MetaOntology. Lastly, section Conclusion and future work concludes this paper and points to important future research.

## Related works

Despite the absence of a well-defined ontology for the Metaverse domain, previous efforts have been made to conceptualize the incorporation of Virtual Reality (VR) in several disciplines. Considering that VR is an integral part of the metaverse, this section examines examples of these efforts. For example, the industrial sector has benefited from incorporating ontology for virtual interactive environments to facilitate interoperability, especially in training and education. In this context, Youcef et al. (2021) developed OntoPhaco, an ontology that provides a formal conceptualization of the Ophthalmology domain. The authors integrated three approaches to evaluate their ontology: criteria-based evaluation, Guizzardi's postulates-based evaluation, and application-based verification. The developed ontology contributed to the VR training and proved utility in allowing students to understand the Phaco technique better. In the same line of research, Dris et al. (2019) designed an ontology for Risk-Hunting training application to enable the trainee to immerse in the work-related educational settings, thereby interoperating the VR with the building information modeling (BIM). The efficiency of the developed ontology was assessed using three sets of questionnaires. The first was carried out before the training to categorize the trainee's technological and knowledge level. The second was conducted following training to gauge knowledge retention. Finally, the trainees received a second copy of the identical questionnaire to capture the resultant knowledge after the training. Developing an ontology to facilitate interoperability for virtual interactive environment in industrial sector has been also addressed in several works, including VISTRA ontology (Gorecky et al., 2014), IVE ontology (Dris et al., 2019), Inoovas ontology (Havard et al., 2017), Ontology for operator training simulator scenarios (Torres Filho et al., 2015), and Ontology for VRSEd project (Walczak et al., 2020).

Semantic web technology and domain ontology were also used to benefit the healthcare domain in VR settings. For example, Baldassini et al. (2017) created an ontology for the Smart Home Simulator project. The aim of the ontology was to customize certain services for older adults by means of VR scenarios. This was achieved by using the ontology to represent users' health conditions, domestic situations, and a variety of comfort indicators, thereby enabling the personalisation of the services provided to specific target users. Another study reported the significance of incorporating an ontology for medical decision support (Heyse et al., 2019). In this study, the authors designed an ontology for VR Exposure Therapy to enable a better service to the target patients and help improve relevant decision support systems. The constructed ontology comprises three layers; (i) Upper ontology: to offer generic and high-level knowledge on the therapy; (ii) Exposure therapy Ontology: which provides a specific conceptualization of the exposure therapy; and (iii) VRET ontology: which applies the exposure therapy in the context of VR. Antoniou et al. (2021) proposed ENTICE ontologyt o enable the extended reality for maximum repurposing capacity. This ontology was able to contextually enclose and annotate any extended reality digital product by connecting medical terminology with UX and educational aspects. An illustration of such a term was given, along with application evidence. The authoring environment and previously proposed visual data structure that could make it easier for non-technical specialists to create and author XR resources can both benefit from the semantic modeling that was established in their work. Incorporating ontology and semantic web technology to offer a better conceptual understanding of domains that interlinks with VR has also been addressed and reported (Mohamad et al., 2021; Asogwa et al., 2022; Dagobert and Helfer, 2022; Narayanasamy et al., 2022).

We intend to extend these efforts by building an ontology that can be extended and interoperated. When dealing with complicated heterogeneous data sources, especially when it comes to an entirely new ecosystem, namely the metaverse, ontologies enable improved data utilization. Additionally, the current stack of ontology tools makes it easier to achieve semantic interoperability by facilitating a more improved user experience. The proposed ontology aims to lessen the learning curve and reduce ontology development. This is by following well-known methodologies in constructing the ontology using popular tools and technological frameworks. Also, the developed ontology uses the most recent RDF and OWL 2 Web Ontology Language specifications from the WWW Consortium, which speeds up the implementation of interoperability. This is also fortified with the use of reasoners and visualization tools to make it easier to accurately translate domain knowledge into a format that machines can understand and interoperate.

## Research methodology

This study incorporates the DSRM approach. This research methodology was designed as a standard paradigm for research conducted in the Information Systems (IS) field to provide a guideline framework for researchers in IS, enabling them to construct artifacts such as constructs, models, methods, and instantiations (von Alan et al., 2004). In the same context, Jones and Gregor (2007) and Venable (2013) contributed to the establishment and formulation of Design Theory. The former presented the structural components that are needed to communicate a design theory, including both core components and additional components. Venable criticized the still-arguable issues pertaining to Design Theory and provided a simplified formulation. Another extensive related study was undertaken by Peffers et al. (2007) who presented their methodology *via* a comprehensive list of activities for any research conducted in the IS discipline. Table 1 demonstrates the activities embedded in this methodology along with their descriptions and steps followed in this research to implement each activity.

**Table 1:** Design science research activities and steps to implement these activities.

**DSR activity**	**Activity description**	**Steps taken to accomplish activity**
Problem identification and motivation	The problem needs to be deconstructed so as to be better understood, and this will assist in the development of a rigorous solution.	- An examination of the literature is conducted to find relevant ontologies, if any. - The literature reveals an absence of ontologies that conceptualize the metaverse domain in terms of embedded technologies.
Define the objectives for a solution	By explicitly defining the quantitative or qualitative nature of the objective.	- The objective of this research is to develop a proof-of-concept ontology that integrates concepts and terminology relevant to state-of-the-art technologies and infrastructures. - This ontology consolidates efforts to better understand the sophistication behind the metaverse and its interrelated aspects.
Design and Development	This is the core part of the research and results in the creation of artifacts and determines the artifacts' architecture and functionality.	- This phase will create an artifact in the form of an ontology (MetaOntology). - The components of the ontology including concepts (classes), attributes (properties), restrictions (facets), and instances (individuals) will be extracted by studying academic and technical resources. - Protégé software is used to design the ontological representation of the ontology components.
Demonstration	The artifact is used to solve a problem, thereby demonstrating its efficacy.	- The proposed methodology is demonstrated by developing a prototype as a proof of concept Ontology. The feasibility of the proposed ontology can be ensured *via* its capacity to offer a better understanding of the metaverse domain and its ecosystem, including embedded technologies and infrastructure.
Evaluation	Certain metrics are used to validate and refine the developed artifacts.	- The designed ontology is evaluated by a domain expert thereby ensuring the correct and factual consepulisaiton of the domain and its underlying structure. - Also, several evaluation metrics were incorporated to evaluate the ontology.
Communication	The artifacts and their importance should be presented to appropriate audiences, including technical personnel.	- Details about the necessity, design approach, and utility of the developed artifact are discussed in this manuscript, leading to information exchange.

## MetaOntology: Ontology design for the metaverse

There are various methodologies that are commonly adopted to design and construct domain ontologies, such as Cyc 101 (Lenat and Guha, 1993), Ontology Development 101 methodology proposed by Stanford University (Noy and McGuinness), YAMO (Dutta et al., 2015), METHONTOLOGY (Fernández-López et al., 1997), and TOVE (Grüninger and Fox, 1995). In this study, we combine METHONTOLOGY (Fernández-López et al., 1997) and Cyc 101 (Lenat and Guha, 1993) to build MetaOntology. The process consists of four key steps, namely (i) determining the domain and scope of the ontology; (ii) ontology reuse; (iii) conceptual model development; and (iv) ontology evaluation. Further, we incorporate “Protégé” software^1^ to implement the ontology. Protégé provides the ability to communicate with other reasoning programs as well as the ability to integrate business rules for inference. Also, Protégé supports the most recent RDF and OWL 2 Web Ontology Language specifications from the WWW Consortium. Next subsections discuss the steps followed to construct MetaOntology.

### Determine the domain and scope of the ontology

This step indicates the specific domain the ontology will conceptualize and the questions the designed ontology will answer. The following are our answers to the questions that are used to determine the domain and scope of the ontology:

#### What is the domain that the ontology will cover?

This study aims to design an ontology (MetaOntology) that conceptualizes the metaverse ecosystem.

#### What is the intention and purpose of this ontology?

The purpose of MetaOntology is to provide an explicit specification and a better understanding of relevant state-of-the-art technologies and infrastructure for the metaverse.

#### Who will use this ontology?

This ontology can be used by academic researchers and industrial practitioners who are interested in finding a conceptualized model for the metaverse technology ecosystem. The proposed ontology can be reused and extended in the future with other concepts, properties, and instances based on the advances in this ecosystem.

#### What are the critical questions that the ontology's embedded information can answer?

∘ What ICT infrastructure, including hardware and software components, are needed for the metaverse?
∘ What are the digitalisation aspects involved in the metaverse?
∘ How is the metaverse different from the typical augmented reality or virtual reality?

### Ontology reuse

Despite limited efforts to offer taxonomies for the metaverse in terms of its ICT infrastructure and relevant technologies, to the best of our knowledge, there has been no attempt to create an ontology for the metaverse devoted to standardizing and formalizing the designated domain knowledge. Therefore, there is no ontology for the metaverse that is publicly available and can be reused.

### Conceptual model development

Developing a conceptual model of an ontology consists of the following steps:

#### Enumerate key terms in the ontology

The critical terms that can be used to formulate the domain and explain the context must be defined. These terms can be categorized into (i) concepts/classes that embody *nouns* standing on their own; (ii) attributes that indicate the physical type of what is being modeled; (iii) properties which are the *verbs* that describe the relationships between concepts and/or attributes of the ontology and their interpretation; and (iv) instances which are the real-life entities of specific classes in ontology.

#### Define classes and class hierarchy

This is a crucial step in ontology engineering because it involves defining the main classes and subclasses (i.e., taxonomy) that frame the skeleton of the conceptual model. Three possible approaches can be followed in this regard; (i) top-down approach: which starts by identifying the most high-level concepts; (ii) bottom-up approach: which starts with the very specific concept definition; and (iii) mixed/combined approach: which integrates concepts captured using both former approaches.

#### Define class properties-slots

The properties in the ontology create the context between various ontology elements and can be categorized into object and datatype properties. The former indicates the connection that can be established between two classes (a subject and object) with a predicate, and the latter embodies the characteristics (physical types) that are associated with the concepts.

#### Define the facets of slots

Each slot is assigned with different facets used to frame its type and value. This includes the physical type (string, integer, Boolean, etc.) and cardinality.

#### Create instances

The final step in the ontology modeling involves populating the resultant ontology with appropriate individual values of each class.

The above indicated steps have been incorporated and followed to design our MetaOntology including concepts, relationships, attributes, and instances. A thorough examination to various academic papers and industrial reports was undergone to extract the technical terminology that is needed to construct the ontology (Kim J, 2021; Lee et al., 2021; Ning et al., 2021; Deloitte., 2022; GlobalData., 2022; Morgan, 2022; Park and Kim, 2022; Seidel et al., 2022; Valaskova et al., 2022; Xu et al., 2022).

In the process of developing MetaOntology, we use Protégé ontology editor for the ontology software modeling, and we use a descriptive language, namely OWL-DL as the logical ontology language. Table 2 lists the main classes of MetaOntology and their descriptions.

**Table 2:** Description of the main classes in MetaOntology.

**Class name**	**Description**
Digitization	The digitisation aspects involved in the metaverse including the communication methods, digital models, digital shadows, virtual overlays, etc.
Technology	Indicates key technologies that derive the development of the Metaverse ecosystem, including Virtual Reality, Augmented Reality, Mixed Reality, Extended Reality, Blockchain, ComputerVision, Edge Computing, IoT, Holographic, etc.
Software component	This includes four generic software categories developed for the Metaverse: scene and object generation, scene and object recognition, software application, and sound and speech recognition.
Hardware component	Indicates the hardware touchpoint devices developed to access the Metaverse, such as VR headset, optical-based devices, on-body-based devices, haptic devices, flying drones, etc.
Metaverse content	Conceptualizes the digital contents that can be developed in the Metaverse virtual environments such as gaming, etc.
Tech company	Technology companies that are involved in the creation of the Metaverse and its ecosystem.
Physical counterpart	The physical counterpart of a certain digital twin.
User feedback	User feedback cues including audio feedback, haptic feedback, and visual feedback.

**Figure 1:**
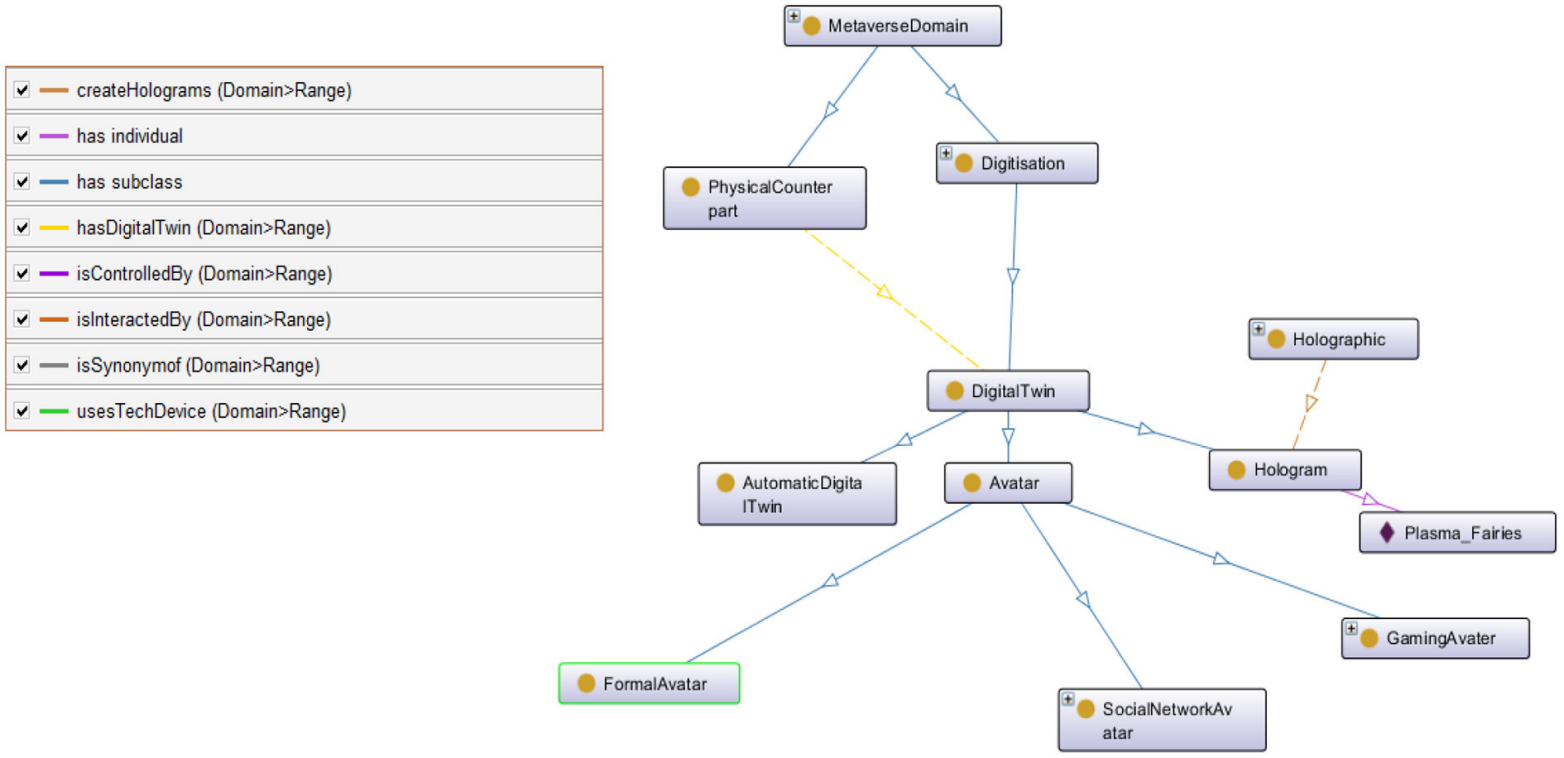
A snapshot of digitaltwin class in MetaOntology.

**Figure 2:**
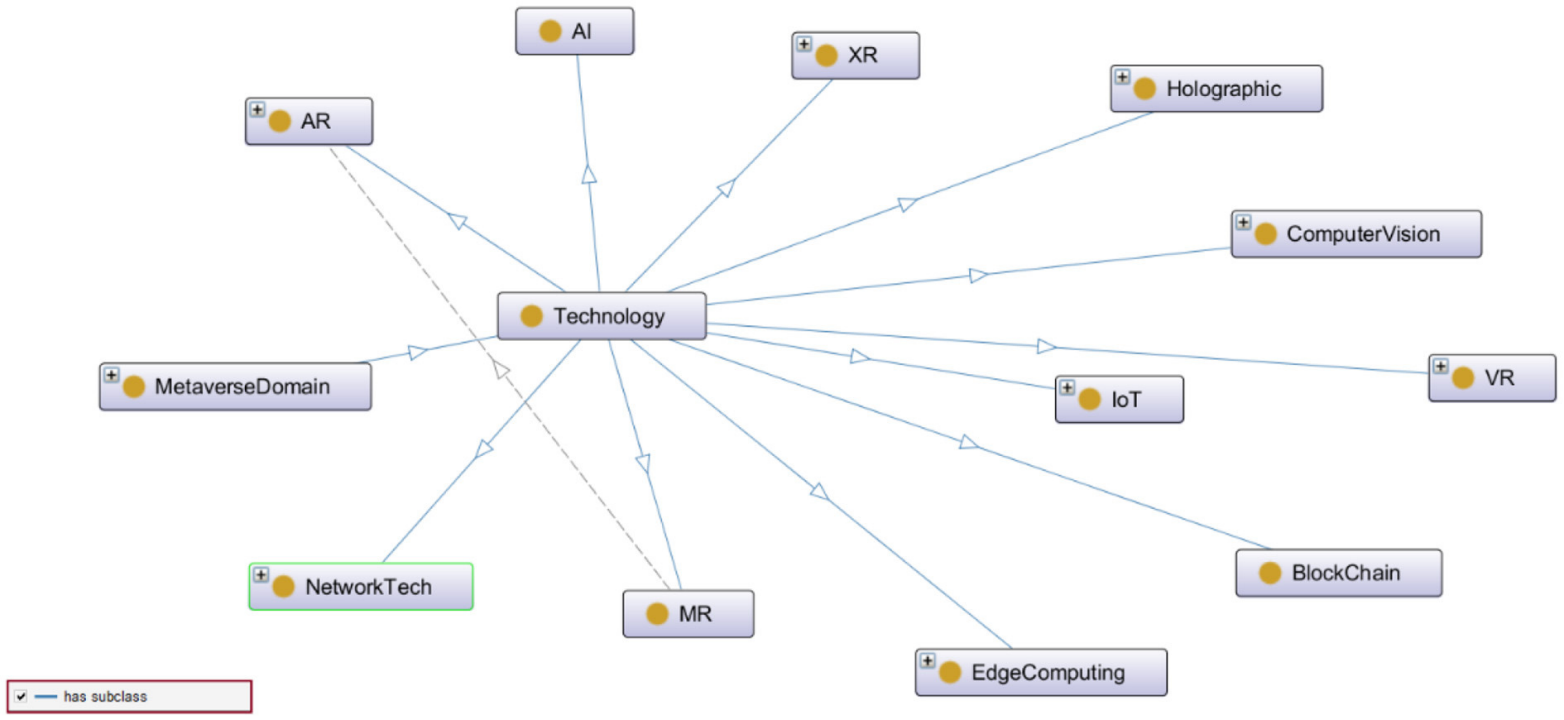
A snapshot of the technology class in MetaOntology.

**Figure 3:**
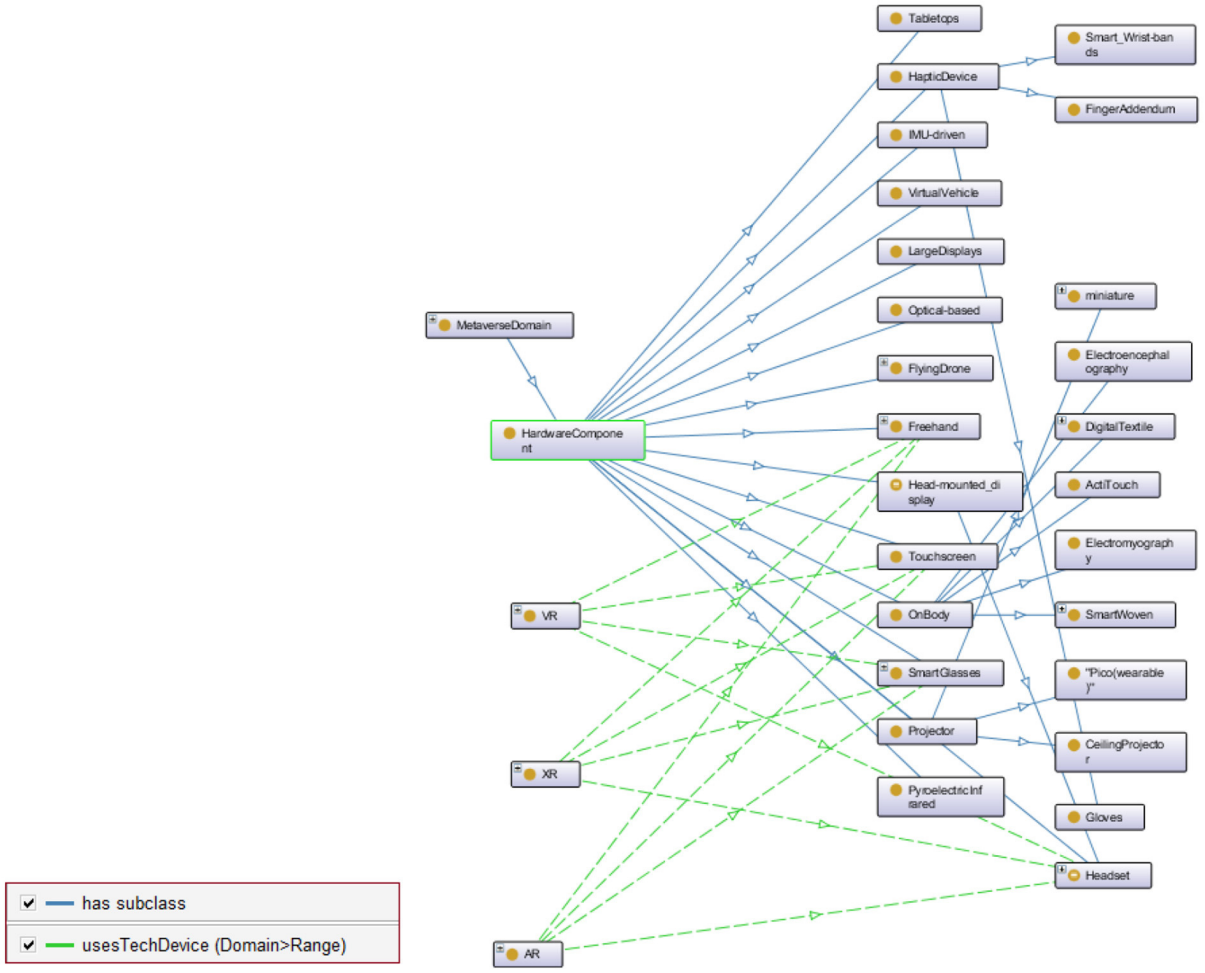
A snapshot of HardwareComponent class in MetaOntology.

To provide detailed conceptualization of the internal classes and the interrelated object and data properties, samples of Metaverse subgraphs are provided and illustrated in Figures 1–3. For example, Figure 1 depicts the DigitalTwin, a subclass of a main class, Digitization. In the digital realm of the Metaverse, a digital twin is a virtual replica of a physical entity or object. This replica can be in the form of an avatar, hologram, or automatic digital twin. Digital twin commonly employs simulation, machine learning, and reasoning to aid decision-making and is updated from real-time data.

Figure 2 demonstrates various technologies incorporated in the Metaverse which are depicted in the figure as subclasses of Technology. These technologies are the backbone of this new ecosystem whereby the physical world can be connected with its digital twin, thus the co-existence of the physical-virtual reality can be attained.

Figure 3 illustrates the HardwareCompnent class and its subclasses. The hardware and software components enable such a parallel universe and provide users with exciting and immersive experiences. The “user experience,” which is the collection of perceptions, feelings, and memories that the user obtains when interacting with the virtual world, is made possible by the interaction between the user and the touchpoint devices or the hardware components.

### Ontology evaluation

The development and the implementation of MetaOntology as discussed in the previous section is followed by ontology evaluation. There are several ontology assessment metrics that have been reported in the literature (Alani and Brewster, 2006; Dellschaft and Staab, 2008; d'Aquin et al., 2009; Yu et al., 2009; Zavitsanos et al., 2010; Ma et al., 2018). Ontology evaluation determines an ontology's quality and if its limitations and criteria have been satisfied. The following subsections discuss various evaluation metrics that are examined and utilized to evaluate MetaOntology.

#### Requirements-oriented evaluation methodology

This study incorporates Yu et al. (2009) evaluation methodology, which stands on five evaluation criteria:

##### Consistency

Reasoning process was followed to make sure MetaOntology is consistent, which is crucial for an ontology's development and testing. Otherwise, no reliable inference may be made. MetaOntology has been reasoned using the FaCT++, HermiT, Pellet, Pellet (Incremental), RacerPro, and TrOWL reasoners to ensure that it is logically consistent. The reasoners examined the class, object, and data property hierarchies, the class/object property assertions, and the ontology's inclusion of the same entities. Consistency checking, concept satisfiability, categorization, and realization are all common inference services that are often offered by a reasoner and are all included in consistency verification by a reasoner. There are no incompatible facts in the MetaOntology.

##### Completeness

This criterion is satisfied when the knowledge depicted by the proposed ontology satisfies the designated domain (Brank et al., 2005). MetaOntology represents the first attempt to conceptualize the metaverse in terms of its underlying infrastructure. The Metaverse is still in its infancy, and various advances are yet to be developed for the metaverse. Hence, MetaOntology can be considered at this stage incomplete and will require to be augmented in the future to ensure completeness.

##### Conciseness

This is to ensure that the developed ontology contains no redundancy. In MetaOntology, the approach followed to incorporate the definitions was crafted to ensure presenting succinct information about the metaverse technologies. Hence, MetaOntology is concise.

##### Expandability

This is to ensure that the developed ontology is designed in a way that can accommodate amendments and augmentation. MetaOntology is created to furnish a capacity to add/alter/delete axioms in the ontology, thereby attaining expandability and interoperability. Also, MetaOntology is aligned with the four extensibility principles, namely ontology term reuse, Ontology semantic alignment, ontology design patterns (ODP) usage for new term generation and existing term editing, and community extensibility (He et al., 2018). Further, we ensure that the proposed ontology offers “as few claims as possible about the world being modelled” (Gruber, 1995), thereby attaining more flexibility and interoperability. Incorporating the minimal ontological commitment criterion following Unified Foundational Ontology (UFL) assists us in building an interoperable ontology *via* solid modeling language, namely OWL 2 which is largely adopted and is supported by the WWW Consortium.

##### Sensitiveness

This aspect points to the vulnerability of the ontology to changes and alterations. MetaOntology is insensitive due to its flexibility and openness to amendments, as discussed in the previous evaluation criterion.

#### Ontology-level evaluation

At the ontology level, there are three evaluation metrics that we used to measure the complexity of the ontology (Srinivasulu et al., 2014; Ajami and Mcheick, 2018):

##### Size of vocabulary

This variable indicates the total number of definitions in the ontology, including classes, individuals, and properties. In particular, the metric can be formulated as:


(1) $$\begin{array}{l} SUV=\;|C_{n}|+|P|+|I_{n}| \end{array}$$

Where *C*_*n*_ denotes the total number of classes, |*P*| represents the number of properties, and |*I*_*n*_| is the number of instances. MetaOntology currently contains 374 components; this indicates that our ontology is relatively small size. Yet, this number is reasonable considering that the proposed ontology is built upon a new domain where new advances are being developed. Hence, the number of definitions will surely increase. Further, the limited size of MetaOntology facilitates interoperability and usability. In particular, ontologies of large vocabularies commonly confront interoperability issues (Hyvönen, 2021) and require significant maintenance time and effort (Sicilia et al., 2012).

##### Edge node ratio

Indicates the density and complexity of the constructed ontology. It is calculated by measuring the ratio between the total count of edges to the total count of nodes. Hence, ENR increases as more edges are added to link nodes, thereby augmenting the ontology's complexity. ENR is computed as follows:


(2) $$\begin{array}{l} ENR=\;\frac{\left|E\right|}{\left|N\right|} \end{array}$$

Where the |*E*| is the number of edges, and |*N*| is the number of nodes in the ontology. The value of MetaOntology' ENR is around ‘1', indicating a modest and straightforward ontology.

##### Tree impurity

Quantifies the degree to which the inheritance hierarchy of an ontology deviates from a tree. It is a logical indication of how effectively inheritance connections are arranged in an ontology. TIP is commonly computed as:


(3) $$\begin{array}{l} TIP=|E^{\prime }|+\;|N^{\prime }|-1 \end{array}$$

Where |*E*′| denotes to the *subclass* edges and |*N*′| is the number of nodes in the inheritance hierarchy of the ontology. The higher the TIP value, the higher complexity the ontology entails, thus making it hard to handle. This is because the inheritance hierarchy of the ontology drifts away from the designated root. TIP of the MetaOntology is <1, which indicates a less complex ontology and implies a relatively modest inheritance hierarchy deviation from the rooted tree.

##### The entropy of ontology graph

This is another criterion to measure the complexity of the ontology. It indicates the number of structural models of the ontology and can be computed as follows:


(4) $$\begin{array}{l} EOG=\;-\;\sum_{i=1}^{n}p\left(i\right)\log_{2}\left(p\left(i\right)\right) \end{array}$$

Where **p(i)** is a probability mass function that indicates the likelihood that a particular class concept has an **i** relation. This function is determined by dividing the degree of the vertex, or the number of edges (or properties) associated with that concept, by the total of all degrees of **V** for each vertex **v** in the graph (relating to a particular concept). In particular, **p(v**_**i**_**)** can be calculated as:


(5) $$\begin{array}{l} p\left(v_{i}\right)=\;\frac{deg\left(v_{i}\right)}{\underset{v\in V}{\sum }deg\left(v\right)} \end{array}$$

The value of EOG for MetaOntology is almost one, indicating that the class structure of MetaOntology is satisfactory.

#### Class-level evaluation

To provide a further evaluation of the ontology's complexity, Brewster et al. (2004) proposed a class-level evaluation metric:

- **The number of classes (NOC)**: The total number of classes in MetaOntology is 90, indicating a relatively adequate ontology. However, this number is likely to be augmented considering that this is a new domain, thus, MetaOntology will be further enriched and populated.
- **The number of properties (NOP)**: The number of properties in MetaOntology is roughly 167, which shows solid reasoning.
- **The number of root classes (NORC)** MetaOntology's NORC is 9, demonstrating diversity in structural ontology design.
- **Relationship richness (RR)** measures the ratio between the count of non-inheritance relationships (i.e., disjoint classes, equivalent classes, and object properties) divided by the sum of subclass relations and non-inheritance relationships. This formula yields a percentage that, disregarding subclass connections over the whole ontology, indicates how much relationship each class has with other classes. RR of MetaOntology is around 0.5, demonstrating richness in terms of knowledge embedded in MetaOntology schema.

#### Ontology usability scale

This study also utilizes Ontology Usability Scale (OUS) (Ma et al., 2018) to further examine the usability of MetaOntology by considering feedback from domain experts. The intuition behind OUS has been derived from the System Usability Scale metric (Brooke, 1996) which is a ten-item Likert scale, with five response options; from Strongly disagree (1) to Strongly agree (5), that is commonly used to establish a metric to measure the usability of products and services. OUS customized SUS and provided a 10-item Likert scale that can be accommodated for ontology usability evaluation. The questions of OUS address three critical usability aspects of designing an ontology, namely the syntax of the ontology (content structure), semantics of the ontology (documentation), and the pragmatic of the ontology (first-hand experience). Table 3 illustrates OUS evaluation questionnaire which includes ten statements and the relevant category of each statement.

**Table 3:** Incorporated ontology usability scale (Ma et al., 2018).

**No**.	**Statement**	**Category**	**Scale (1 2 3 4 5)**
1	The purpose of this ontology is clear.	Semantics	
2	I need more examples than provided in the documentation to make sure how to use the ontology.	Pragmatics	
3	I found the concepts and relations in this ontology properly described in natural language.	Semantics	
4	There is an inconsistency between the formal specification of concepts and relations in this ontology and their descriptions in natural language.	Semantics/Syntax	
5	I would imagine that most domain experts would understand this ontology very quickly.	Semantics	
6	I think that I would need the support of a person experienced with this ontology to be able to use it.	Pragmatics	
7	I am confident I understand the conceptualization of ontology.	Semantics	
8	The attributes in this ontology fail to describe the concepts properly.	Syntax	
9	I think the relations in this ontology relate to appropriate concepts.	Semantics	
10	I think the class hierarchy of this ontology needs better organization.	Semantics/Syntax	

OUS evaluation metric is used in this study whereby the ontology is given to three independent evaluators with experience in both VR technicalities and ontology design. After examining MetaOngology, the OUS questionnaire is provided to each evaluator to obtain feedback on the usability of constructed ontology. The answers to the questionnaire are obtained, and the response for each statement is averaged using the following formula:


(6) $$\begin{array}{l} AS_{m}=\;\frac{\overset{r}{\underset{i=1}{\sum }}R_{m}^{i}}{r} \end{array}$$

**Figure 4:**
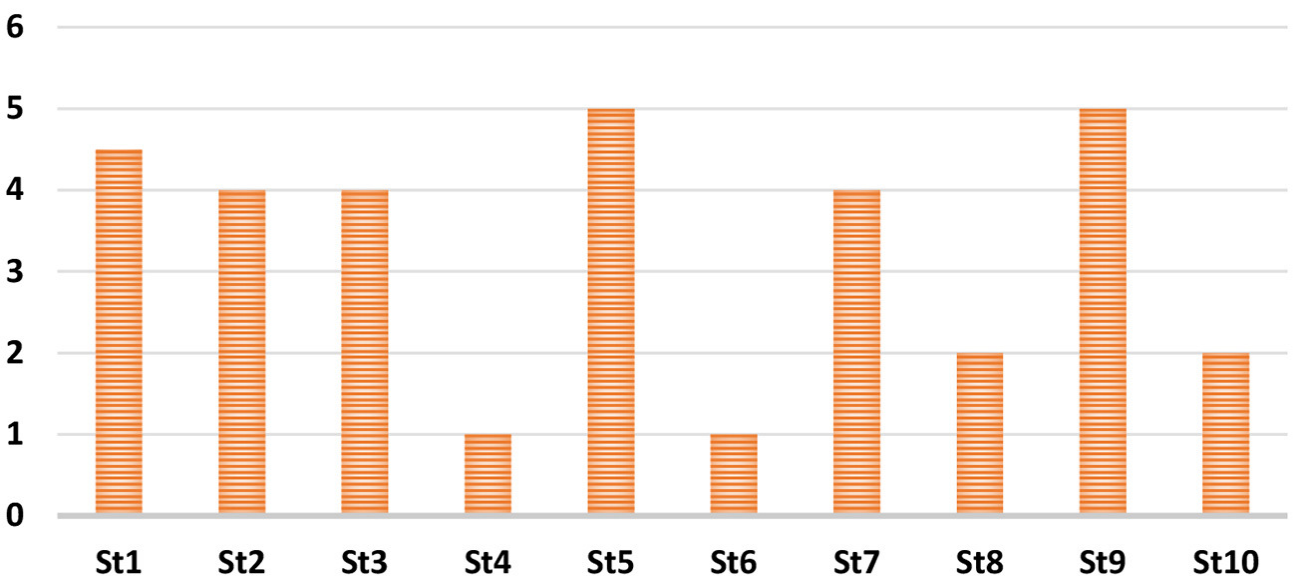
The OUS score average for each statement provided in Table 3.

Where *AS*_*m*_ is the average of scores retrieved by each evaluator for a statement (*m*), *r* is the number of evaluators, and $R_{m}^{i}$ is the score obtained by the evaluator (*R*) for a certain statement (*m*). Figure 4 demonstrates the scoring average that is calculated for each statement as depicted in Table 3 after obtaining the relevant response from the evaluators.

As can be observed from Figure 4, the evaluators exhibit satisfaction with the MetaOntology in terms of its semantics, syntax, and pragmatics. This is evident as the evaluators agreed strongly with the statements that indicate a positive form of the ontology (i.e., st1, st2, st3, st5, st7, and st9). Also, the evaluators disagreed and strongly disagreed with statements that indicate a negative form of the ontology (i.e., st4, st6, st8, and st10). This evaluation metric verifies the usability of MetaOntology in representing the designated domain.

## Conclusion and future work

The metaverse has the potential to disrupt various aspects of our life. Users in this new ecosystem will be able to access 3D virtual or augmented reality settings using virtual reality headsets, digital glasses, smartphones, and other gadgets. As technology converges, more mergers and synergies could take place. In order to better grasp the structure of information already spread throughout this inventive ecosystem, it is necessary to develop a common language, nomenclature, and modeling representations of this new domain, including dimensions of cutting-edge technology. This paper presents MetaOntology, an explicit specification of relevant underlying state-of-the-art technologies and infrastructure of the metaverse. A systematic approach is followed to design, implement, and evaluate MetaOntology. Due to the immaturity of the metaverse, MetaOntology does not provide the whole picture. Therefore, we will keep augmenting, updating, and polishing MetaOntology, thereby furnishing a topical representation of the domain. Further, a domain-specific knowledge graph will be constructed upon the underlying schematic structure of MetaOntology. This new knowledge graph can then be used to integrate the metaverse's heterogeneous data sources into a unified knowledge base and applied to downstream tasks, thus proving another utility of the constructed ontology and its usability.

## Data availability statement

The raw data supporting the conclusions of this article will be made available by the authors, without undue reservation.

## Author contributions

The author confirms being the sole contributor of this work and has approved it for publication.

## Conflict of interest

The author declares that the research was conducted in the absence of any commercial or financial relationships that could be construed as a potential conflict of interest.

## Publisher's note

All claims expressed in this article are solely those of the authors and do not necessarily represent those of their affiliated organizations, or those of the publisher, the editors and the reviewers. Any product that may be evaluated in this article, or claim that may be made by its manufacturer, is not guaranteed or endorsed by the publisher.

## Footnotes

^1^https://protege.stanford.edu/.
